# Transcriptional Analysis of Metabolic Pathways and Regulatory Mechanisms of Essential Oil Biosynthesis in the Leaves of *Cinnamomum camphora* (L.) Presl

**DOI:** 10.3389/fgene.2020.598714

**Published:** 2020-11-12

**Authors:** Jiexi Hou, Jie Zhang, Beihong Zhang, Xiaofang Jin, Haiyan Zhang, Zhinong Jin

**Affiliations:** ^1^Jiangxi Provincial Engineering Research Center for Seed-Breeding and Utilization of Camphor Trees, The School of Hydraulic and Ecological Engineering, Nanchang Institute of Technology, Nanchang, China; ^2^Key Laboratory of Silviculture, Co-Innovation Center of Jiangxi Typical Trees Cultivation and Utilization, College of Forestry, Jiangxi Agricultural University, Nanchang, China

**Keywords:** transcriptional analysis, terpenoid biosynthesis, chemotypes, leaves, *Cinnamomum camphora*

## Abstract

The roots, bark, and leaves of *Cinnamomum camphora* are rich in essential oils, which mainly comprised monoterpenes and sesquiterpenes. Although the essential oils obtained from *C. camphora* have been widely used in pharmaceutical, medicinal, perfume, and food industries, the molecular mechanisms underlying terpenoid biosynthesis are poorly understood. To address this lack of knowledge, we performed transcriptome analysis to investigate the key regulatory genes involved in terpenoid biosynthesis in *C. camphora*. High-oil-yield trees of linalool type and low-oil-yield trees were used to assemble a *de novo* transcriptome of *C. camphora*. A total of 121,285 unigenes were assembled, and the total length, average length, N50, and GC content of unigenes were 87,869,987, 724, 1,063, and 41.1%, respectively. Comparison of the transcriptome profiles of linalool-type *C. camphora* with trees of low oil yield resulted in a total of 3,689 differentially expressed unigenes, among them 31 candidate genes had annotations associated with metabolism of terpenoids and polyketides, including four in the monoterpenoid biosynthesis pathway and three in the terpenoid backbone biosynthesis pathway. Collectively, this genome-wide transcriptome provides a valuable tool for future identification of genes related to essential oil biosynthesis. Additionally, the identification of a cohort of genes in the biosynthetic pathways of terpenoids provides a theoretical basis for metabolic engineering of essential oils in *C. camphora*.

## Introduction

As a member of the Lauraceae family, *Cinnamomum camphora* is an evergreen tree widely distributed around the southern portions of the Yantze River in China, with the largest numbers in Jiangxi, Guangdong, Fujian, Zhejiang, Yunnan, and Sichuan. Many parts of the camphor tree, including roots, stems, leaves, flowers, fruits, and bark, are rich in essential oils that have a wide range of uses in the pest control, spice, cosmetics, and pharmaceutical industries (Guo et al., 2016; Jiang et al., 2016). In order to achieve sustainable production, large-scale plantations have been established in China. Under this model, camphor trees are maintained as bushes, and the leaves and twigs are harvested every year for essential oil production. *C. camphora* plantations are characterized by fast growth and high afforestation density, and therefore, they can provide a large number of raw materials for the essential oil industry.

Essential oils in the camphor tree primarily consist of monoterpenes and sesquiterpenes. Depending on the principal chemical component of their essential oils, camphor trees can be divided into different chemical types, such as linalool, borneol, camphor, cineol, and iso-nerolidol (Guo et al., 2017). With the gradual improvements in *C. camphora* cultivation and essential oil extraction techniques, use of *C. camphora* as a source of natural linalool, which has long been preferred over its synthetic form for the perfume industry, is becoming more popular. However, the molecular mechanisms underlying the terpenoid biosynthesis in *C. camphora* are still poorly understood, which impedes the progress of identifying “superior” *C. camphora* clones that could increase the efficiency of using *C. camphora* for essential oil production.

The biosynthetic pathway of terpenoids in plants starts with the synthesis of the universal terpenoid precursors isopentenyl diphosphate (IPP) and dimethylallyl diphosphate (DMAPP) through the mevalonate (MVA) or the methylerythritol 4-phosphate (MEP) pathways. Isoprenyl diphosphate synthases (IDSs) catalyze reactions involving IPP and DMAPP, forming the intermediate phosphate precursors, geranyl diphosphate (GPP), geranylgeranyl diphosphate (GGPP), and farnesyl diphosphate, which are then catalyzed by terpene synthases (TPSs) to form diverse terpenoids (Chen et al., 2003; Cheng et al., 2007; Ma et al., 2012). The enzymes in the MVA pathway have been shown to be located in cytosol or peroxisomes, whereas all the enzymes involved in the MEP pathway are found in plastids (Reumann et al., 2007; Simkin et al., 2011). In contrast, the localizations of IDSs and TPSs are more diverse and are often at the site of terpenoid biosynthesis (Ma et al., 2012). Although characterizations of the genes involved in terpenoid biosynthesis have been reported in many plant species, including peppermint, *Arabidopsis*, conifers, *Hevea brasiliensis*, and *Salvia miltiorrhiza* (Battaile and Loomis, 1961; Lange and Ghassemian, 2003; Sando et al., 2008a,b; Zulak and Bohlmann, 2010; Ma et al., 2012), many of the enzyme-encoding genes have yet to be elucidated because of the complexity of the terpenoid biosynthesis pathway, especially in the species without a whole-genome sequence.

In the studies of *C. camphora*, transcriptome analyses for leaves of camphor trees of linalool-, borneol-, camphor-, cineol-, and iso-nerolidol types led to the identification of 424 unigenes in the biosynthetic pathways of terpenoids (Jiang et al., 2014) and transcriptome profiling of linalool and borneol types of *C. camphora* identified three monoterpene synthase genes possibly involved in the biosynthesis of borneol (Chen et al., 2018). Although these two transcriptome studies have provided valuable insights into the terpenoid biosynthesis pathway, comparisons between only high-oil-yielding individuals could exclude the identification of genes upstream of the biosynthetic pathway of terpenoids. In this study, we used RNA-Seq to identify key enzyme-encoding genes putatively associated with terpenoid biosynthesis in *C. camphora*, which may partially determine its essential oil composition. Differentially expressed genes (DEGs) between camphor trees of linalool-type and low-oil-yield camphor trees were identified. In addition to four unigenes in the “monoterpenoid biosynthesis” pathway, this analysis also identified three unigenes in the “terpenoid backbone biosynthesis” pathway. Overall, these results contribute to the general understanding of terpenoid biosynthesis in *C. camphora* and provide a theoretical basis for metabolic engineering of terpenoids in this species.

## Materials and Methods

### Plant Materials

Mature leaves of linalool-type and low-oil-yield *C. camphora* were harvested from 3-year-old clones grown at the experimental garden of NanChang Institute of Technology, China, in October. The clones were propagated from mother trees through cutting propagation. For each biological replicate (*n* = 3), leaves from at least five tree clones, which were cloned from the same mother tree, were collected and mixed. The three replicates each for the low-oil-yield variety and linalool-type *C. camphora* were termed N- (1–3) and LI- (1–3) separately. All the samples were harvested and snap-frozen in liquid nitrogen and then stored at −80°C until RNA extraction. Meanwhile, leaves were also collected for oil extraction and chemical component determination.

### Essential Oil Extraction and Determination of Essential Oil Yield

Fresh leaves were harvested and processed for oil extraction immediately after harvesting. Leaf samples were hydrodistilled in a modified Clevenger-type apparatus for 4 h. Extracted essential oils were weighed, and oil yields were calculated using the formula:

Essential oil yield (%)=W1/W2×100

where W1 is the weight of extracted essential oil, and W2 is the weight of fresh leaves.

### Gas Chromatography–Mass Spectrometry Analysis

Analyses of essential oils were performed on a gas chromatography system (Agilent 7890B, United States) equipped with a TG-5MS capillary column (30 m × 0.25-mm internal diameter × 0.25-μm film thickness). Helium with a flow rate of 1 mL/min was used as the carrier gas. The injector was maintained at 220°C. The initial temperature of the column was set at 40°C and increased to 280–300°C at a rate of 6.5°C/min. The mass spectrometer was set to scan in the range of 50–650 m/z with a scan rate of 0.5 scans/s. Compounds were determined based on their relative retention time and mas spectra data. Details were described in our previous study (Zhang et al., 2019).

### cDNA Library Preparation and RNA Sequencing

Total RNA was extracted from samples using the RNeasy Plant Mini Kit (Qiagen, United States) following the manufacturer’s instructions. Next, gDNA was removed using DNase I digestion (Qiagen, United States). The integrity of extracted RNA was determined by the RNA Nano 6000 Assay Kit of the Agilent 2100 Bioanalyzer (Agilent Technologies, United States). Extracted RNA was sent to Gene *Denovo* Biotechnology Co. (Guangzhou, China) for cDNA library preparation and sequencing. Up to 1 μg of total RNA from each sample was used for library construction using the NEB #7530 kit for Illumina^®^ (New England Biolabs, United States), as described by the manufacturer. mRNA was first enriched by oligo(dT) beads and fragmented into small fragments using fragmentation buffer and reverse-transcribed into cDNA with ProtoScript II Reverse Transcriptase and random primers. Second-strand cDNA was synthesized by DNA polymerase I along with RNase H, dNTP, and buffer. Next, cDNA fragments were purified with 1.8 × Agencourt AMPure XP Beads, end-repaired, and ligated to Illumina sequencing adapters after addition of poly(A) tails. Ligated fragments were subjected to size selection by gel electrophoresis and polymerase chain reaction (PCR) amplified. The resulting cDNA library was sequenced using Illumina HiSeq^TM^ 4000.

### *De novo* Assembly and Functional Annotation

Raw reads from each sample were filtered by removing reads containing more than 10% of unknown nucleotides, low-quality reads that contained more than 40% of low-quality (*Q* value ≤ 20) bases, and adapter sequences according to the Illumina adapter list. Residual ribosome RNA (rRNA) reads were identified by mapping the filtered reads to rRNA, and the identified sequences were then removed. The resulting 87.9 M reads were assembled using short read assembler, Trinity (Grabherr et al., 2011), and further clustered to obtain non-redundant unigenes by Corset (Davidson and Oshlack, 2014). The completeness of the transcriptomic assembly was assessed using BUSCO (Simao et al., 2015) by comparing the 1,440 embryophyta-specific genes to our unigenes. Unigenes were annotated by BLASTx (with an *E*-value threshold of 1e^–5^) to the Swiss-Prot protein database (Bairoch and Apweiler, 2000) and NCBI non-redundant protein (Nr) database (O’Leary et al., 2016). Nr annotation results of unigenes were used for Gene Ontology (GO) annotation by Blast2GO software (Conesa et al., 2005), and functional classification of unigenes was performed using WEGO software (Ye et al., 2006).

### Identification of Differentially Expressed Genes

Expression values of each gene were calculated as reads per kilobase (of exon) per million (RPKM) mapped reads. Biological samples with low and high oil contents were grouped, and comparison was undertaken to identify DEGs between the two experimental groups. Principal component analysis (PCA) was also performed as a quality control. DEGs with a fold change ≥ 2 and *p* < 0.01 across the two experimental groups were identified using the edgeR package (Robinson et al., 2010).

### Quantitative Reverse Transcriptase–PCR

Leaves from five randomly selected 3-year-old tree clones of each chemotype were collected for quantitative reverse transcriptase (RT)–PCR analysis. Total RNA was extracted and reverse-transcribed into cDNA using the HiScript II Q RT SuperMix for quantitative PCR (qPCR) (Vazyme, China). The PCRs were performed using ChamQTM SYBR^®^ qPCR Master Mix (Vazyme, China) according to the manufacturer’s instructions on an ABI Step One Plus System with three replicates. Sequences of the gene-specific primers are listed in Supplementary Table S1. Relative gene expression was calculated using the 2^–△△*Ct*^ method, and *ACTIN* was chosen as the internal reference gene to normalize expression levels.

## Results

### Chemical Composition of Leaf Essential Oils From *C. camphora* of Different Oil Contents

In general, there were no major differences in morphology between camphor trees of different chemotypes, and the trees were therefore initially classified by odor alone. Fresh leaves of the six samples were harvested, and the essential oils were obtained by water distillation. The average oil outputs of the samples from linalool- and low-oil-yield chemotypes were 1.3 and 0.3%, respectively. Chemical composition of the extracted essential oils was determined by gas chromatography–mass spectrometry (GC-MS). Table 1 lists the major components detected, 1–15 are monoterpenes, and the remaining are sesquiterpenes. In the essential oils of the linalool type, β-linalool was the principal constituent, making up ∼90% in average. Among the various constituents of the essential oils extracted from the different chemotypes of camphor trees, the major difference was observed for the monoterpene content. Therefore, transcriptome analyses between trees of linalool type and trees with low oil yield were performed to investigate the molecular mechanisms underlying the biosynthesis of monoterpenes.

**TABLE 1:** Chemical composition of the essential oils from leaf extracts of *C. camphora*.

No.	Compound	N-1 (%)	N-2 (%)	N-3 (%)	LI-1(%)	LI-2(%)	LI-3 (%)
1	α-Thujene	1.51	—	—	—	0.1	—
2	Pinene	8.28	4.63	—	—	0.36	0.19
3	Camphene	—	1.49	—	—	—	0.14
4	Phellandrene	26.19	0.88	—	0.19	0.12	0.47
5	β-Myrcene	2.26	—	—	0.16	0.16	—
6	D-Limonene	—	—	—	0.15	0.26	0.26
7	Eucalyptol	37.7	2.14	—	0.59	—	0.46
8	β-Ocimene	0.56	4.87	—	—	—	0.68
9	Terpinene	3.48		—	—	—	—
10	β-Linalool	—	2.06	—	90.38	86.8	89.16
11	Borneol acetate	—	1.95	—	—	—	—
12	Camphor	—	—	—	—	1.36	—
13	Terpineol	10.57	—	—	—	0.13	0.07
14	Citronellol	—	—	—	—	0.13	—
15	Citral	—	—	—	0.43	1.16	0.54
16	Caryophyllene	2.2	13.16	8.58	2.07	1.71	1.03
17	Alloaromadendrene	—	1.24	0.18	—	—	—
18	Germacrene D	1.37	1.28	3.11	1.26	0.24	0.59
19	Naphthalene	—	1.36		—	—	—
20	Humulene	1.76	3.41	5.49	—	—	0.59
21	ç-Elemene	1.25	15.01	4.43	0.86	3.36	1.18
22	Nerolidol	—	22.2	44.95	0.11	0.14	0.24
23	(−)-Spathulenol	—	1.76	3.72	—	0.94	0.41
24	Nerolidyl acetate	—	1.29	—	—	—	—
25	Guaiol	0.9	—	0.24	—	—	—
26	Caryophyllene oxide	—	1.17	1.1	0.28	0.34	0.58
27	Others and Unknown	1.97	20.1	28.2	3.52	2.69	3.41

**N: C. camphora with low oil yield; LI: C. camphora of linalool type; “—” represents a compound not detected or in trace amounts (< 0.1%).**

### *De novo* Assembly of the *C. camphora* Transcriptome

To construct a genome-wide transcriptome of *C. camphora*, total RNA of the experimental samples was isolated. This pooled total RNA was sequenced and a total of 87.9 M 150 bp paired-end reads remained after raw reads were processed with sequence filtering and quality control (Supplementary Table S2). *De novo* assembly was carried out with short reads using Trinity (see section “Materials and Methods”). A total of 121,285 unigenes were assembled with a high N50 value (1063), as well as high values for both the maximum unigene length (15,713) and percentage of mapped reads (>98%), indicating a good assembly quality (Table 2). In addition, BUSCO analysis was performed to assess the completeness of the final genome assembly. Based on the 1,440 embryophytic-specfic genes, 79.8% were identified as complete genes, including 76.3% complete and single-copy genes and 3.5% complete and duplicated genes, and 8.8% were identified as fragmented genes, indicating good completeness of the genome assembly (Supplementary Figure S1).

**TABLE 2:** Statistics for *de novo* assembly of the *C. camphora* transcriptome.

Total assembled bases	87.9M
Total no. of unigenes	121,285
N50 (bp)	1,063
Shortest unigene length (bp)	201
Longest unigene length (bp)	15,713
Average unigene length (bp)	724
Mapped reads (%)	>98%

### Functional Annotation of Unigenes

**FIGURE 1:**
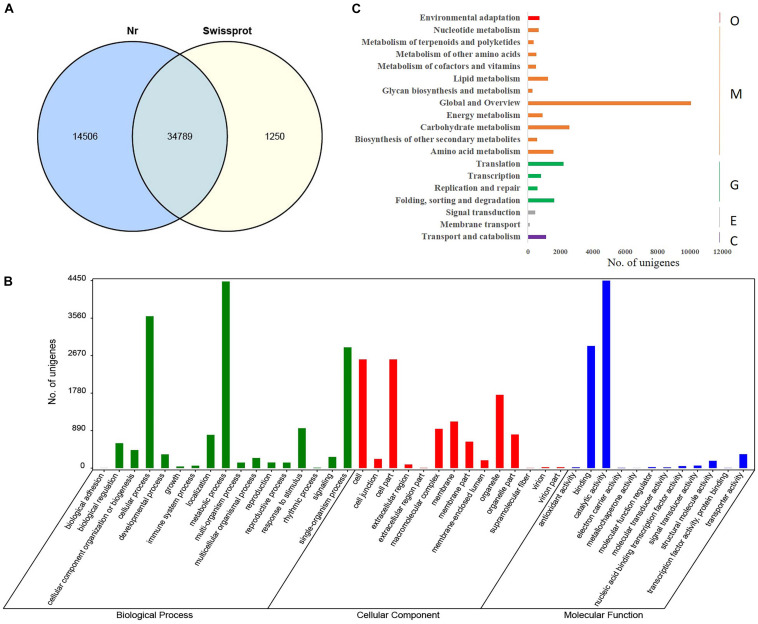
Functional annotation and GO classification of assembled unigenes in *C. camphora*. **(A)** Functional annotation of assembled unigenes by BLASTx against Nr and Swissprot databases with an *E*-value threshold of 1e^–5^. A total of 34,789 unigenes were commonly annotated in the two databases. **(B)** GO terms of *C. camphora* unigenes determined by Blast2GO fell into the three categories of biological process, cellular component, and molecular function, where metabolic process, cell part, and catalytic activity were the three most prevalent subcategories, respectively. **(C)** KEGG classification of annotated unigenes. The *x-*axis shows the number of unigenes annotated, and the *y* axis shows the KEGG metabolic pathways the unigenes fell into, including organismal system (O), metabolism (M), genetic information processing (G), environmental information processing (E), and cellular processes (C).

Functional annotation of 121,285 *C. camphora* unigenes was performed by comparing unigene sequences to the protein databases Nr and Swissprot with an *E*-value threshold of 1e^–5^ using BLASTx analysis. This analysis yielded a total of 50,545 annotated genes, with 34,789 unigenes in common between two databases (Figure 1A). A total of 49,295 unigenes had significant hits in the Nr database. Further GO annotation analysis assigned the 49,295 unigenes to three main categories, including biological process, cellular component, and molecular function (Figure 1B). Within the biological process, “metabolic process” was highly represented (29.5%) followed by “cellular process” (24.1%). Within the cellular component, “cell” (23.7%), “cell part” (23.7%), and “organelle” (16.0%) were most represented. Similarly, “catalytic activity” and “binding” were the most dominant terms under molecular function, accounting for 55.4 and 36.1%, respectively (Supplementary Table S3). To systematically analyze the metabolic pathways of unigene products in cells and predict the function of these unigene products, Kyoto Encyclopedia of Genes and Genomes (KEGG) analysis was performed, and 11,111 unigenes were annotated and classified into 19 metabolic pathways. The category “Global and Overview” included the largest number of unigenes (Figure 1C), among which “metabolic pathways” and “biosynthesis of secondary metabolites” were dominant, accounting for 40.8 and 22.2%, respectively (Supplementary Table S4). Additionally, 359 unigenes were placed into the category “metabolism of terpenoids and polyketides,” which included “terpenoid backbone biosynthesis” (32.3%), “monoterpenoid biosynthesis” (5.0%), “diterpenoid biosynthesis” (13.4%), “sesquiterpenoid and triterpenoid biosynthesis” (5.0%), “carotenoid biosynthesis” (15.3%), “brassinosteroid biosynthesis” (8.9%), “zeatin biosynthesis” (8.4%), and “limonene and pinene degradation” (11.7%) (Supplementary Table S4).

### RNA-Seq Analyses of DEGs Between *C. camphora* Leaves With High and Low Oil Contents

**FIGURE 2:**
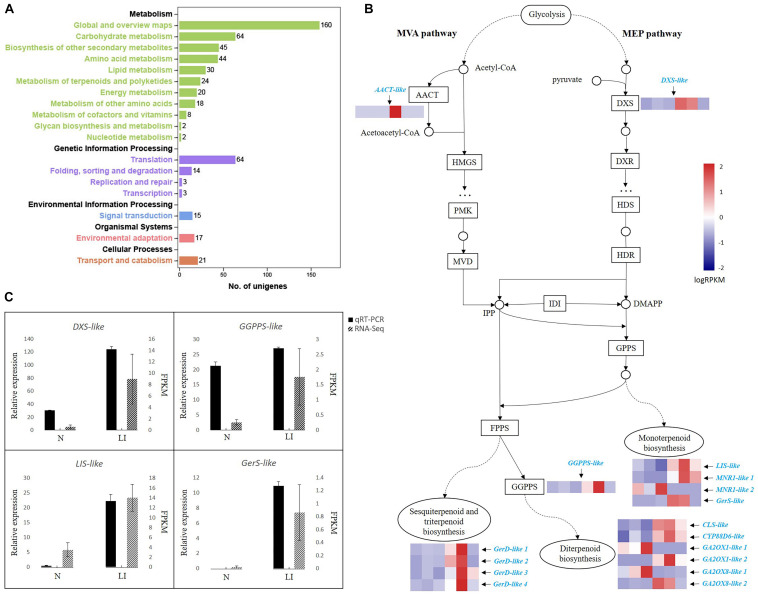
Transcriptional cascade of DEGs in terpenoid backbone biosynthesis in linalool-type *C. camphora*. **(A)** KEGG pathway annotations of DEGs. The *x-*axis indicates the number of unigenes annotated in each corresponding pathway. The five categories of KEGG pathways are divided and color-coded. **(B)** DEGs in terpenoid backbone biosynthesis. The transcriptional cascade of terpenoid backbone biosynthesis is shown in the form of protein-coding enzymes or nodes annotated by KEGG pathway analysis. Enzyme expression patterns are indicated with logRPKM values, and DEGs are in azure. The left three and right three columns represent low oil content type (N) and linalool type (LI), respectively. AACT, acetyl-CoA C-acetyltransferase; DXS, 1-deoxy-D-xylulose-5-phosphate synthase; HMGS, hydroxymethylglutaryl-CoA synthase; PMK, phosphomevalonate kinase; MVD, mevalonate diphosphate decarboxylase; DXR, 1-deoxy-d-xylulose-5-phosphate reductoisomerase; HDS, hydroxymethylbutenyl 4-diphosphate synthase; HDR, (E)-4-hydroxy-3-methyl-but-2-enyl diphosphate reductase; IDI, isopentenyl-diphosphate delta-isomerase; GPPS, geranyl diphosphate synthase; FPPS, farnesyl pyrophosphate synthase; GGPPS, geranylgeranyl pyrophosphate synthase; LIS, linalool synthase; MNR1, (+)-neomenthol dehydrogenase-like isoform; GerS, geraniol synthase; GerD, (−)-germacrene D synthase; CLS, ent-copalyl diphosphate synthase; CYP88D6, cytochrome P450 88D6; GA2OX, Gibberellin 2-beta-dioxygenase. **(C)** qRT-PCR validation of RNA-Seq data. The black bars represent relative gene expression determined by qRT-PCR (left *y*-axis) and the dotted gray bars represent gene expression levels detected by RNA-Seq (right *y-*axis). Standard errors of the three technical replicates of each sample of the qRT-PCR analysis and three biological replicates in the two experimental groups of the RNA-Seq analysis are indicated by error bars.

To get a better understanding of the dynamic processes of terpenoid biosynthesis in *C. camphora*, we performed transcriptome analyses of camphor leaves with high and low oil contents based on the oil yield and GC-MS results (Table 1). Trimmed reads were mapped to the *de novo* transcriptome assembly of *C. camphora*. PCA demonstrated a good similarity between samples in the high- and low-oil-content groups (Supplementary Figure S2A). Similarly, the range of Pearson correlation coefficient between two samples in each experimental group is 0.93–0.96 (Supplementary Figure S2B), also indicating that the samples in the same groups are highly correlated. Further transcriptome analysis identified 3,689 DEGs with *p* < 0.01 and |log2 fold_change| > 2, which comprised 2,120 up-regulated and 1,569 down-regulated unigenes. The GO terms “metabolic process” in the “biological process” category and “catalytic activity” in the “molecular function” category were the two most highly represented within DEGs (Supplementary Figure S3). In accordance with the GO analysis, KEGG enrichment analysis revealed that most of the 3,689 DEGs were in “metabolism” class (Figure 2A), which included “biosynthesis of secondary metabolites,” “phenylpropanoid biosynthesis,” “monoterpenoid biosynthesis,” “terpenoid backbone biosynthesis,” and other pathways (Supplementary Table S5).

### Putative Genes Involved in Terpenoid Backbone and Terpenoid Biosynthesis in *C. camphora*

Annotated protein-coding enzymes were mapped into the terpenoid backbone biosynthesis pathway, and gene expression data of each enzyme in the two experimental groups were indicated with logRPKM values (Figure 2B and Supplementary Table S6). Two unigenes encoding key enzymes, *AACT-like* in the MVA pathway and *DXS-like* in the MEP pathway, exhibited a higher expression level in the groups with high oil contents. Because both MVA and MEP pathways generate the precursors IPP and DMAPP for the final production of terpenoids, increased expression of these two genes could explain the higher amount of terpenoids we observed in this group (Table 1). Additionally, the *GGPPS-like* gene also had a higher expression level in the leaves of linalool type. Among the DEGs, 14 unigenes were annotated as terpenoid synthase–encoding genes, including four in monoterpenoid biosynthesis, four in sesquiterpenoid and triterpenoid biosynthesis, and six in diterpenoid biosynthesis (Figure 2B and Supplementary Table S6). One *LIS-like* and one *GerS-like* unigene were up-regulated in the monoterpenoid biosynthesis pathway, indicating that increased availability of terpenoid precursors, along with increased levels of monoterpene synthases, might be the reason for the higher oil yield and higher accumulation of linalool and other monoterpenes in the trees of linalool type. Besides monoterpenoid synthases, four sesquiterpenoid synthase unigenes encoding GerD and four diterpenoid synthase unigenes encoding CLS, CYP88D6, GA2OX1, and GA2OX8 were also up-regulated. In general, the upregulation of terpenoid synthase unigenes in the trees of linalool-type *C. camphor* is consistent with their increased accumulation of terpenoids, suggesting that the presence of various terpenoid synthases might be the reason for the occurrence of varied terpenoids in the essential oils of *C. camphora*. To validate the reliability of our RNA-Seq data, four DEGs were chosen for qRT-PCR examination. As shown in Figure 2C, the expression profiles of these unigenes observed by qRT-PCR are identical to their profiles identified in the RNA-Seq analysis.

## Discussion

*C. camphora* oils have a wide variety of uses, including ornamental, medicinal, and industrial, which makes understanding the regulation of essential oil biosynthesis in camphor tree highly important (Guo et al., 2016; Jiang et al., 2016; Chen et al., 2018). Because of earlier unregulated harvesting, many natural sources of camphor trees have been exhausted. In response to this, camphor tree plantations have been established in many countries including China and Japan. These camphor trees are maintained as bushes and are harvested for twigs and leaves before essential oil extraction. Camphor trees can be divided into different chemotypes based on their main terpenoid components. In this study, we identified camphor trees of linalool type and low oil contents without a dominant terpene component. A total of 26 main components were identified by GC-MS. We further demonstrated that the chemical components in the essential oils of *C. camphora* leaf extracts contained mainly monoterpenes and sesquiterpenes with very minor quantities of diterpenes (Table 1). Moreover, linalool took up ∼90 of the total essential oil contents in linalool type, indicating that monoterpene levels account for the major difference between the essential oils of different chemotypes.

Terpenoid biosynthesis and its regulation are extensively documented in many plant species. Despite having high structural diversity, all terpenes are derived from two isomeric basic backbone molecules, IPP and DMAPP, which are synthesized either through the MVA or MEP pathways (Sacchettini and Poulter, 1997). Although many members of the Lauraceae family are rich in terpenoids and are economically important tree species, limited studies are available analyzing genes involved in its terpenoid biosynthesis pathway (Yang et al., 2005; Lin et al., 2014; Chen et al., 2018; Chen et al., 2020). This study was focused on identifying genes involved in terpenoid biosynthesis and key enzymes involved in the biosynthesis of the backbone IPP and DMAPP molecules in *C. camphora*. A transcriptome of *C. camphora* was also generated to provide a reference for future RNA-Seq–based expression profiling of terpenoid biosynthesis. Functional annotation analysis showed significant enrichment in genes associated with “metabolic pathways” and “biosynthesis of secondary metabolites” (Figure 1). This is consistent with our observation that September–November is the time of the year when *C. camphora* harbors the highest oil contents (Qin et al., 2015), and it is also the harvesting time of our samples. Furthermore, 116 and 18 expressed unigenes were identified to be highly homologous with 49 and 11 known enzymes in the terpenoid backbone biosynthesis and monoterpenoid biosynthesis pathways, respectively.

By comparing the transcriptome profiles of leaves of linalool and leaves with low oil contents, 3,689 unigenes were identified as DEGs. Among them, expression levels of *AACT-like* and *DXS-like*, catalyzing the first steps toward IPP and DMAPP in the MVA and MEP pathways, were up-regulated in the leaves of linalool type. *DXS* genes are believed to be a significant control point in the MEP pathway (Wolfertz et al., 2004; Rohdich et al., 2006), and manipulation of gene expression levels of *DXS* in *Arabidopsis* altered the levels of tocopherols, carotenoids, chlorophylls, abscisic acid, and various other isoprenoids (Estévez et al., 2001). However, the contribution of each pathway to the generation of the common precursors for varied terpenoid biosynthesis is still unknown, because crosstalk between the two pathways has been documented in many species including chamomile, *Arabidopsis*, tobacco, and snapdragon (Adam and Zapp, 1998; Bick and Lange, 2003; Hemmerlin et al., 2003; Laule et al., 2003; Dudareva et al., 2005). In addition, the expression level of *GGPPS-like* in the “terpenoid backbone biosynthesis” pathway was up-regulated in the comparison, indicating increased supply of precursors for diterpenes biosynthesis. Although the percentage of diterpenes in leaf extracts of linalool-type camphor trees is not high, their oil yield is much higher than trees of low oil yield. Therefore, higher *GGPPS* expression level may simply be correlated with a general increase in oil content. Interestingly, it has been documented that GPP, the precursor for monoterpene, is constitutively and ubiquitously expressed in *Arabidopsis* (Van Schie et al., 2013), and our results also showed that the level of *GPPS* was indifferent between the groups regardless of different accumulation of monoterpenes.

Noticeably, *LIS-like* shared a high sequence identity (>99%) with some of the identified linalool synthases in the Lauraceae family. The conserved DDxxD and NSE/DTE motifs for Mg^2+^ or Mn^2+^ ion binding as well as the predicted D562 active site cleft were all properly distributed in the LIS-like protein (Supplementary Figure S4 and Supplementary Table S7). However, caution should be used when assigning protein functions to terpenoid synthase genes predicted based on sequence similarities as it has been reported that point mutations alone were sufficient to significantly change the kinetic activity of a linalool synthase gene in *Cinnamomum osmophloeum* (Lin et al., 2014). Additionally, alternative splicing has been shown to alter linalool biosynthesis, as reported in *Camellia sinensis* (Liu et al., 2018). Higher levels of monoterpene production could potentially lower production of other terpenes and isoprenoid derivatives due to the competition for common precursors. Indeed, four unigenes in the “carotenoid biosynthesis pathway” and two unigenes in the “steroid biosynthesis pathway” were down-regulated in the comparison (Supplementary Table S6). Similar examples can be seen in tobacco plants producing monoterpene where lower levels of β-caryophyllene were detected in the flowers of these plants (Lücker et al., 2004). Additionally, both transgenic tomato and *Arabidopsis* plants producing a phytoene synthase and the strawberry linalool/nerolidol synthase separately had significant growth retardation due to the severe inhibition of gibberellin biosynthesis (Fray et al., 1995; Aharoni et al., 2004).

Other genes of interest emerged from this study, which include several members of the WRKY family. *WRKY21-like*, *WRKY47-like*, and *WRKY65-like* were all up-regulated in the leaves with high oil content (Supplementary Table S6). WRKY transcription factors have been reported to regulate the expression of genes involved in terpene biosynthesis in many species, including *Arabidopsis*, cotton, *Artemisia annua*, *Catharanthus roseus*, and others (Xu et al., 2004; Ma et al., 2009; Mao et al., 2011; Suttipanta et al., 2011). Moreover, it was suggested that the linalool-type *C. osmophloeum* contains a W-box element (T)(T)TGAC(C/T) in the promoter region of the linalool synthase gene, enabling regulation by WRKY transcription factors (Lin et al., 2014). Based on these previous studies, we suspect that members of the WRKY family might be involved in the regulation of terpene synthase genes of *C. camphora* and therefore could potentially play critical roles in the quantitative difference of oil contents between different chemotypes.

All in all, this study identified a cohort of genes in the terpenoid backbone biosynthesis and monoterpenoid biosynthesis pathways that were commonly up-regulated in leaves with high linalool contents compared to leaves with low oil contents. The knowledge obtained from this study could facilitate manipulation of camphor tree essential oil production through metabolic engineering of essential oil biosynthesis. Additional studies should focus on the isolation of monoterpene synthases genes from different chemotypes of *C. camphora*. Additionally, functional analyses are needed to demonstrate the impact of these genes on terpenoid production.

## Data Availability Statement

Raw reads yielded from Illumina sequencing have been uploaded to the NCBI Sequence Read Archive (https://www.ncbi.nlm.nih.gov/sra) and accession numbers for the six samples are as below: N-1 (SRR11362618); N-2 (SRR11362617); N-3 (SRR12576812); LI-1 (SRR11362614); LI-2 527 (SRR11362613); and LI-3 (SRR12576811).

## Author Contributions

HZ, JH, and ZJ designed the experiments. BZ, JZ, XJ, and JH performed the experiments. HZ and JH analyzed the data. JH wrote the manuscript. All authors read and approved the final manuscript.

## Conflict of Interest

The authors declare that the research was conducted in the absence of any commercial or financial relationships that could be construed as a potential conflict of interest.

## Supplementary Material

Supplementary Figure 1 BUSCO analysis of the final transcriptomic assembly.

Supplementary Figure 2 Relationship between the samples.

Supplementary Figure 3 GO enrichment histogram of DEGs.

Supplementary Figure 4 Amino acid sequence alignment of identified LIS-like in *C. camphora* with linalool synthase CoLIS-LL in *C. osmophloeum* (accession number: JX028232).

Supplementary Table 1 Sequences of the gene-specific primers used in quantitative RT-PCR.

Supplementary Table 2 Raw transcriptomic data.

Supplementary Table 3 GO enrichment analysis of *C. camphora* unigenes.

Supplementary Table 4 KEGG enrichment of *C. camphora* unigenes.

Supplementary Table 5 KEGG enrichment of DEGs.

Supplementary Table 6 Putative unigenes associated with terpenoid biosynthesis.

Supplementary Table 7 Sequences of putative unigenes associated with terpenoid biosynthesis.

## References

[B1] Adam K.-P. Zapp J. (1998). Biosynthesis of the isoprene units of chamomile sesquiterpenes. *Phytochemistry* 48 953–959. 10.1016/S0031-9422(97)00992-8

[B2] Aharoni A. Giri A. P. Verstappen F. W. A. Bertea C. M. Sevenier R. Sun Z. (2004). Gain and loss of fruit flavor compounds produced by wild and cultivated strawberry species. *Plant Cell* 16 3110–3131. 10.1105/tpc.104.023895 15522848 PMC527202

[B3] Bairoch A. Apweiler R. (2000). The SWISS-PROT protein sequence database and its supplement TrEMBL in 2000. *Nucleic Acids Res.* 28 45–48. 10.1093/nar/28.1.45 10592178 PMC102476

[B4] Battaile J. Loomis W. D. (1961). Biosynthesis of terpenes: II. The site and sequence of terpene formation in peppermint. *BBA* 51 545–552. 10.1016/0006-3002(61)90612-6 13865784

[B5] Bick J. A. Lange B. M. (2003). Metabolic cross talk between cytosolic and plastidial pathways of isoprenoid biosynthesis: unidirectional transport of intermediates across the chloroplast envelope membrane. *Arch. Biochem. Biophys.* 415 146–154. 10.1016/s0003-9861(03)00233-9 12831836

[B6] Chen C. Zheng Y. Zhong Y. Wu Y. Li Z. Xu L.-A. (2018). Transcriptome analysis and identification of genes related to terpenoid biosynthesis in *Cinnamomum camphora*. *BMC Genomics* 19:550. 10.1186/s12864-018-4941-1 30041601 PMC6057064

[B7] Chen F. Tholl D. D’Auria J. C. Farooq A. Pichersky E. Gershenzon J. (2003). Biosynthesis and emission of terpenoid volatiles from *Arabidopsis* flowers. *Plant Cell* 15 481–494. 10.1105/tpc.007989 12566586 PMC141215

[B8] Chen Y. Li Z. Zhao Y. Gao M. Wang J. Liu K. (2020). The Litsea genome and the evolution of the laurel family. *Nat. Commun.* 11:1675. 10.1038/s41467-020-15493-5 32245969 PMC7125107

[B9] Cheng A.-X. Lou Y.-G. Mao Y.-B. Lu S. Wang L.-J. Chen X.-Y. (2007). Plant terpenoids: biosynthesis and ecological functions. *J. Integr. Plant Biol.* 49 179–186. 10.1111/j.1744-7909.2007.00395.x

[B10] Conesa A. Götz S. García-Gómez J. M. Terol J. Talón M. Robles M. (2005). Blast2GO: a universal tool for annotation, visualization and analysis in functional genomics research. *Bioinformatics* 21 3674–3676. 10.1093/bioinformatics/bti610 16081474

[B11] Davidson N. M. Oshlack A. (2014). Corset: enabling differential gene expression analysis for de novo assembled transcriptomes. *Genome Biol.* 15:410. 10.1186/s13059-014-0410-6 25063469 PMC4165373

[B12] Dudareva N. Andersson S. Orlova I. Gatto N. Reichelt M. Rhodes D. (2005). The nonmevalonate pathway supports both monoterpene and sesquiterpene formation in snapdragon flowers. *Proc. Nat. Acad. Sci. U.S.A.* 102 933–938. 10.1073/pnas.0407360102 15630092 PMC545543

[B13] Estévez J. M. Cantero A. Reindl A. Reichler S. León P. (2001). 1-Deoxy-D-xylulose-5-phosphate synthase, a limiting enzyme for plastidic isoprenoid biosynthesis in plants. *J. Biol. Chem.* 276 22901–22909. 10.1074/jbc.M100854200 11264287

[B14] Fray R. G. Wallace A. Fraser P. D. Valero D. Hedden P. Bramley P. M. (1995). Constitutive expression of a fruit phytoene synthase gene in transgenic tomatoes causes dwarfism by redirecting metabolites from the gibberellin pathway. *Plant J.* 8 693–701. 10.1046/j.1365-313X.1995.08050693.x

[B15] Grabherr M. G. Haas B. J. Yassour M. Levin J. Z. Thompson D. A. Amit I. (2011). Full-length transcriptome assembly from RNA-Seq data without a reference genome. *Nat. Biotechnol.* 29 644–652. 10.1038/nbt.1883 21572440 PMC3571712

[B16] Guo S. Geng Z. Zhang W. Liang J. Wang C. Deng Z. (2016). The chemical composition of essential oils from *Cinnamomum camphora* and their insecticidal activity against the stored product pests. *Int. J. Mol. Sci.* 17:1836. 10.3390/ijms17111836 27827929 PMC5133837

[B17] Guo X. Cui M. Deng M. Liu X. Huang X. Zhang X. (2017). Molecular differentiation of five *Cinnamomum camphora* chemotypes using desorption atmospheric pressure chemical ionization mass spectrometry of raw leaves. *Sci. Rep.* 7:46579. 10.1038/srep46579 28425482 PMC5397862

[B18] Hemmerlin A. Hoeffler J.-F. Meyer O. Tritsch D. Kagan I. A. Grosdemange-Billiard C. (2003). Cross-talk between the cytosolic mevalonate and the plastidial methylerythritol phosphate pathways in tobacco bright yellow-2 cells. *J. Biol. Chem.* 278 26666–26676. 10.1074/jbc.M302526200 12736259

[B19] Jiang H. Wang J. Song L. Cao X. Yao X. Tang F. (2016). GC×GC-TOFMS Analysis of essential oils composition from leaves, twigs and seeds of Cinnamomum camphora L. Presl and their insecticidal and repellent activities. *Molecules* 21 423–423. 10.3390/molecules21040423 27043503 PMC6274170

[B20] Jiang X. Wu Y. Xiao F. Xiong Z. Xu H. (2014). Transcriptome analysis for leaves of five chemical types in *Cinnamomum camphora*. *Yi chuan* 36 58–68. 10.3724/sp.j.1005.2014.00058 24846919

[B21] Lange B. M. Ghassemian M. (2003). Genome organization in Arabidopsis thaliana: a survey for genes involved in isoprenoid and chlorophyll metabolism. *Plant Mol. Biol.* 51 925–948. 10.1023/a:1023005504702 12777052

[B22] Laule O. Fürholz A. Chang H.-S. Zhu T. Wang X. Heifetz P. B. (2003). Crosstalk between cytosolic and plastidial pathways of isoprenoid biosynthesis in *Arabidopsis thaliana*. *PNAS* 100 6866–6871. 10.1073/pnas.1031755100 12748386 PMC164538

[B23] Lin Y.-L. Lee Y.-R. Huang W.-K. Chang S.-T. Chu F.-H. (2014). Characterization of S-(+)-linalool synthase from several provenances of *Cinnamomum osmophloeum*. *Tree Genet Genomes* 10 75–86. 10.1007/s11295-013-0665-1

[B24] Liu G.-F. Liu J.-J. He Z.-R. Wang F.-M. Yang H. Yan Y.-F. (2018). Implementation of CsLIS/NES in linalool biosynthesis involves transcript splicing regulation in *Camellia sinensis*. *Plant Cell Environ.* 41 176–186. 10.1111/pce.13080 28963730

[B25] Lücker J. Schwab W. van Hautum B. Blaas J. van der Plas L. H. W. Bouwmeester H. J. (2004). Increased and altered fragrance of tobacco plants after metabolic engineering using three monoterpene synthases from lemon. *Plant Physiol.* 134 510–519. 10.1104/pp.103.030189 14718674 PMC316330

[B26] Ma D. Pu G. Lei C. Ma L. Wang H. Guo Y. (2009). Isolation and characterization of AaWRKY1, an Artemisia annua transcription factor that regulates the amorpha-4,11-diene synthase gene, a key gene of artemisinin biosynthesis. *Plant Cell Physiol.* 50 2146–2161. 10.1093/pcp/pcp149 19880398

[B27] Ma Y. Yuan L. Wu B. Li X. E. Chen S. Lu S. (2012). Genome-wide identification and characterization of novel genes involved in terpenoid biosynthesis in *Salvia miltiorrhiza*. *J. Exp. Bot.* 63 2809–2823. 10.1093/jxb/err466 22291132 PMC3346237

[B28] Mao G. Meng X. Liu Y. Zheng Z. Chen Z. Zhang S. (2011). Phosphorylation of a WRKY transcription factor by two pathogen-responsive MAPKs drives phytoalexin biosynthesis in *Arabidopsis*. *Plant Cell* 23 1639–1653. 10.1105/tpc.111.084996 21498677 PMC3101563

[B29] O’Leary N. A. Wright M. W. Brister J. R. Ciufo S. Haddad D. McVeigh R. (2016). Reference sequence (RefSeq) database at NCBI: current status, taxonomic expansion, and functional annotation. *Nucleic Acids Res.* 44 D733–D745. 10.1093/nar/gkv1189 26553804 PMC4702849

[B30] Qin Z.-H. Li J.-F. Shi Y. Lu K.-C. Tang F.-C. Liu H.-L. (2015). Content and main compositions analysis of essential oil in branches and leaves of *Cinnamomum camphora* in different months (in Chinese). *Guangxi For. Sci.* 44 428–430. 10.19692/j.cnki.gfs.2015.04.021

[B31] Reumann S. Babujee L. Ma C. Wienkoop S. Siemsen T. Antonicelli G. E. (2007). Proteome analysis of *Arabidopsis* leaf peroxisomes reveals novel targeting peptides, metabolic pathways, and defense mechanisms. *Plant Cell* 19 3170–3193. 10.1105/tpc.107.050989 17951448 PMC2174697

[B32] Robinson M. D. McCarthy D. J. Smyth G. K. (2010). edgeR: a Bioconductor package for differential expression analysis of digital gene expression data. *Bioinformatics* 26 139–140. 10.1093/bioinformatics/btp616 19910308 PMC2796818

[B33] Rohdich F. Lauw S. Kaiser J. Feicht R. Köhler P. Bacher A. (2006). Isoprenoid biosynthesis in plants – 2C-methyl-d-erythritol-4-phosphate synthase (IspC protein) of *Arabidopsis thaliana*. *FEBS J.* 273 4446–4458. 10.1111/j.1742-4658.2006.05446.x 16972937

[B34] Sacchettini J. C. Poulter C. D. (1997). Creating isoprenoid diversity. *Science* 277 1788–1789. 10.1126/science.277.5333.1788 9324768

[B35] Sando T. Takaoka C. Mukai Y. Yamashita A. Hattori M. Ogasawara N. (2008a). Cloning and characterization of mevalonate pathway genes in a natural rubber producing plant, *Hevea brasiliensis*. *Biosci. Biotechnol. Biochem.* 72 2049–2060. 10.1271/bbb.80165 18685207

[B36] Sando T. Takeno S. Watanabe N. Okumoto H. Kuzuyama T. Yamashita A. (2008b). Cloning and characterization of the 2-C-methyl-D-erythritol 4-phosphate (MEP) pathway genes of a natural-rubber producing plant, *Hevea brasiliensis*. *Biosci. Biotechnol. Biochem.* 72 2903–2917. 10.1271/bbb.80387 18997428

[B37] Simao F. A. Waterhouse R. M. Ioannidis P. Kriventseva E. V. Zdobnov E. M. (2015). BUSCO: assessing genome assembly and annotation completeness with single-copy orthologs. *Bioinformatics* 31 3210–3212. 10.1093/bioinformatics/btv351 26059717

[B38] Simkin A. J. Guirimand G. Papon N. Courdavault V. Thabet I. Ginis O. (2011). Peroxisomal localisation of the final steps of the mevalonic acid pathway in planta. *Planta* 234:903. 10.1007/s00425-011-1444-6 21655959

[B39] Suttipanta N. Pattanaik S. Kulshrestha M. Patra B. Singh S. K. Yuan L. (2011). The transcription factor CrWRKY1 positively regulates the terpenoid indole alkaloid biosynthesis in *Catharanthus roseus*. *Plant Physiol.* 157 2081–2093. 10.1104/pp.111.181834 21988879 PMC3327198

[B40] Van Schie C. C. N. Haring M. A. Schuurink R. C. (2013). “Prenyldiphosphate synthases and gibberellin biosynthesis,” in *Isoprenoid Synthesis in Plants and Microorganisms: New Concepts and Experimental Approaches*, eds Bach T. Rohmer M. (New York: Springer), 214–220.

[B41] Wolfertz M. Sharkey T. D. Boland W. Kühnemann F. (2004). Rapid regulation of the methylerythritol 4-Phosphate pathway during isoprene synthesis. *Plant Physiol.* 135 1939–1945. 10.1104/pp.104.043737 15286290 PMC520765

[B42] Xu Y.-H. Wang J.-W. Wang S. Wang J.-Y. Chen X.-Y. (2004). Characterization of GaWRKY1, a cotton transcription factor that regulates the sesquiterpene synthase gene (+)-delta-cadinene synthase-A. *Plant Physiol.* 135 507–515. 10.1104/pp.104.038612 15133151 PMC429402

[B43] Yang T. Li J. Wang H.-X. Zeng Y. (2005). A geraniol-synthase gene from *Cinnamomum tenuipilum*. *Phytochemistry* 66 285–293. 10.1016/j.phytochem.2004.12.004 15680985

[B44] Ye J. Fang L. Zheng H. Zhang Y. Chen J. Zhang Z. (2006). WEGO: a web tool for plotting GO annotations. *Nucleic Acids Res.* 34 W293–W297. 10.1093/nar/gkl031 16845012 PMC1538768

[B45] Zhang B. Wu C. Xiao Z. Zhang H. Cao M. Liu Y. (2019). Chemical constituents and chemotypes of fresh leaf essential oil of wild species belonging to Sect. Camphor (Trew.) Meissn. in southeastern China. *J. Essent.* 22 1115–1122. 10.1080/0972060X.2019.1662331

[B46] Zulak K. G. Bohlmann J. (2010). Terpenoid biosynthesis and specialized vascular cells of conifer defense. *J. Integr. Plant Biol.* 52 86–97. 10.1111/j.1744-7909.2010.00910.x 20074143

