# Supplementary material for: Transcriptional Analysis of Metabolic Pathways and Regulatory Mechanisms of Essential Oil Biosynthesis in the Leaves of Cinnamomum camphora (L.) Presl
Source: Front Genet. 2020 Nov 12;11:598714. doi: 10.3389/fgene.2020.598714 (PMC7689033; doi:10.3389/fgene.2020.598714)
Supplement: Supplementary Figure 1 — BUSCO analysis of the final transcriptomic assembly. [file Data_Sheet_1.docx]

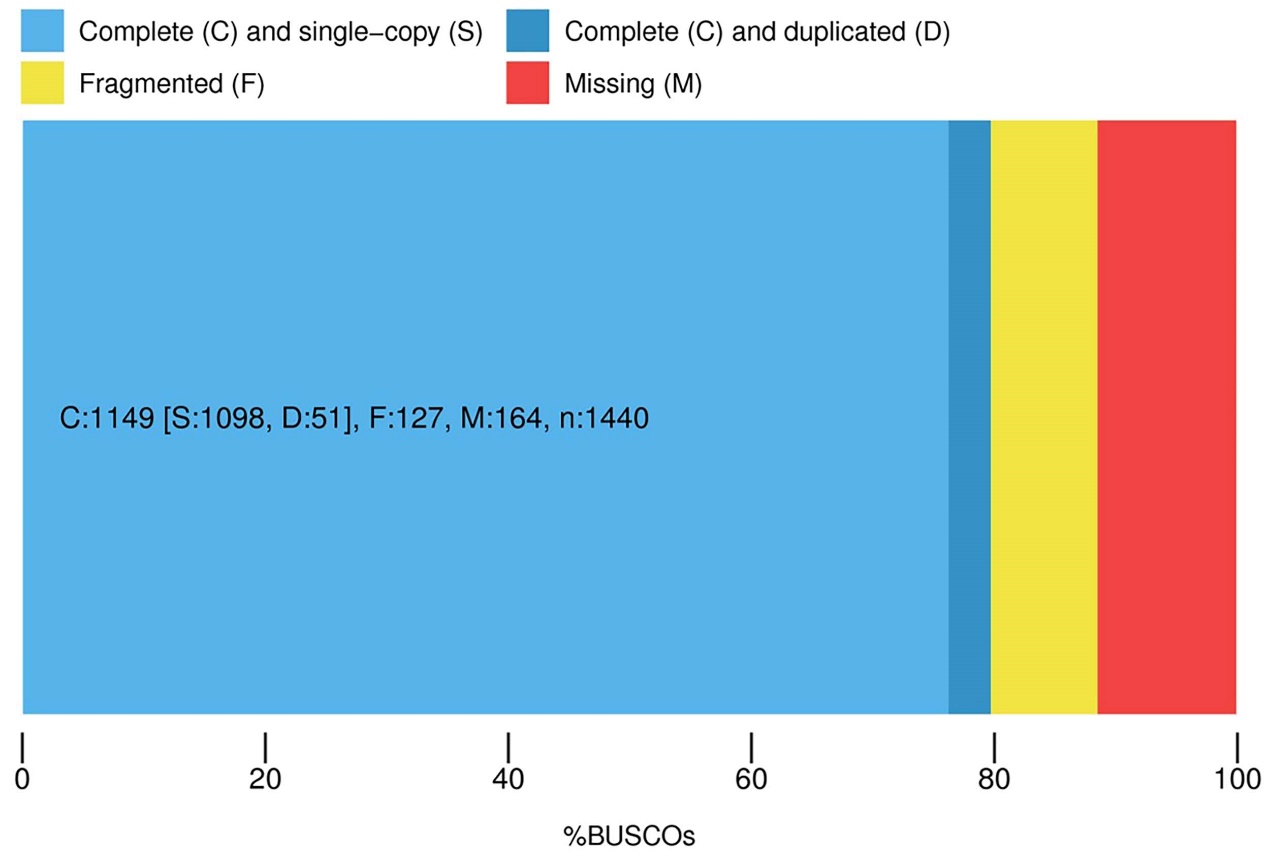


**Figure S1.**  BUSCO analysis of the final transcriptomic assembly. Out of the 1,440 embryophytic-specific genes, 79.8% (76.3% complete and single-copy genes and 3.5%complete and duplicated genes) and 8.8% were identified as complete and fragmented genes, separately.


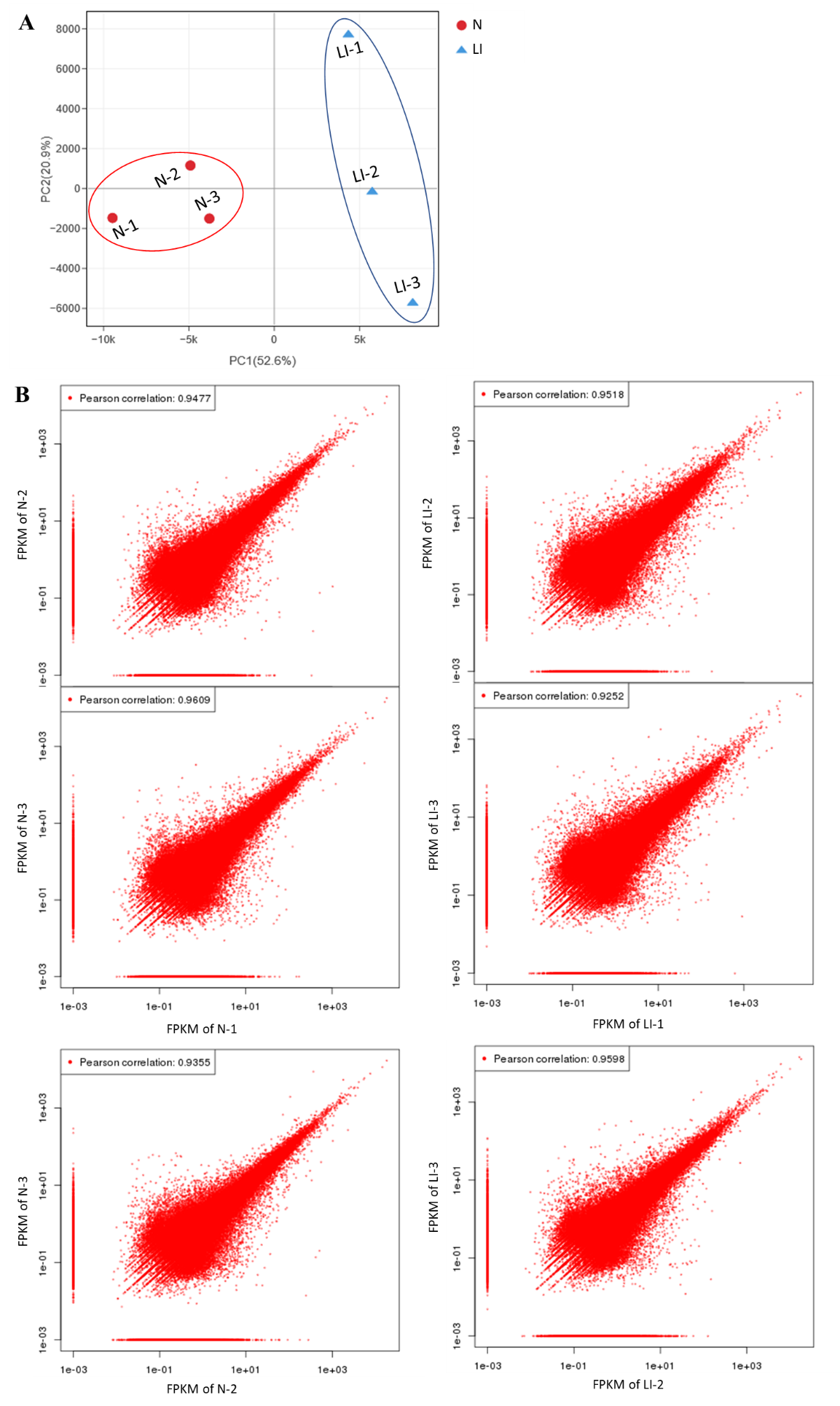


**Figure S2.** Relationship between the samples. (a) PCA analysis of the transcriptome profiles of the six samples. (b) Statics of Pearson correlation between two samples in each experimental group.


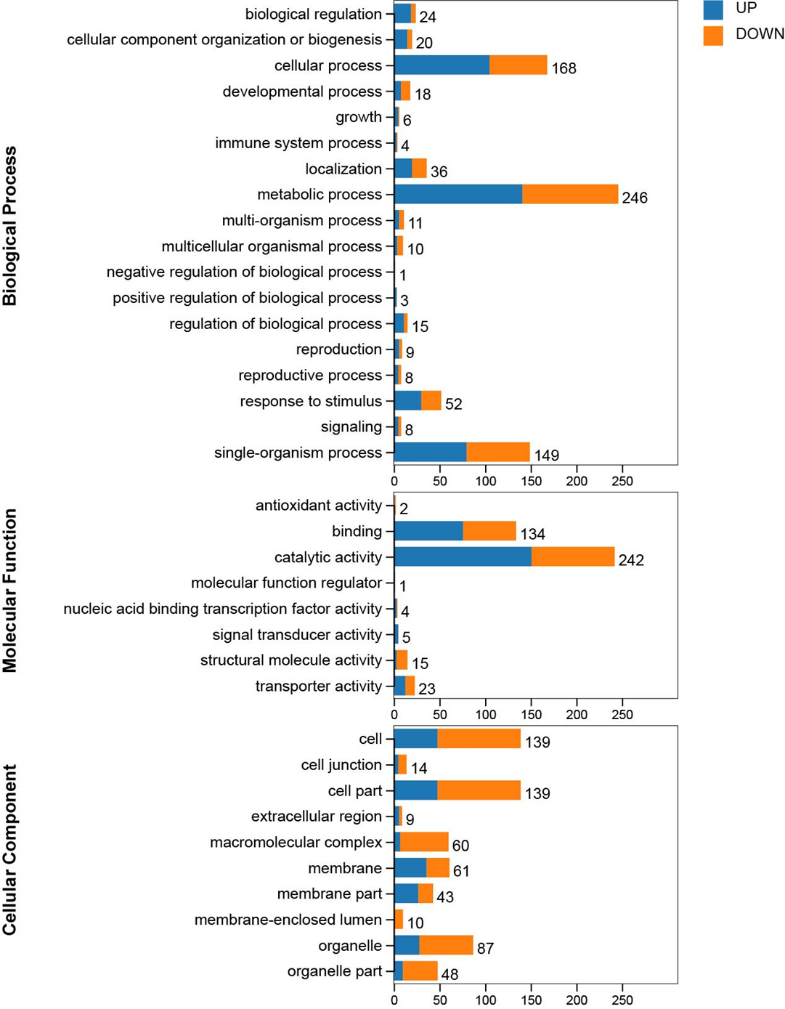

**Figure S3.** GO enrichment histogram of DEGs. GO terms are classified into three categories “Biological Process”, “Cellular component”, and “Molecular Function”.


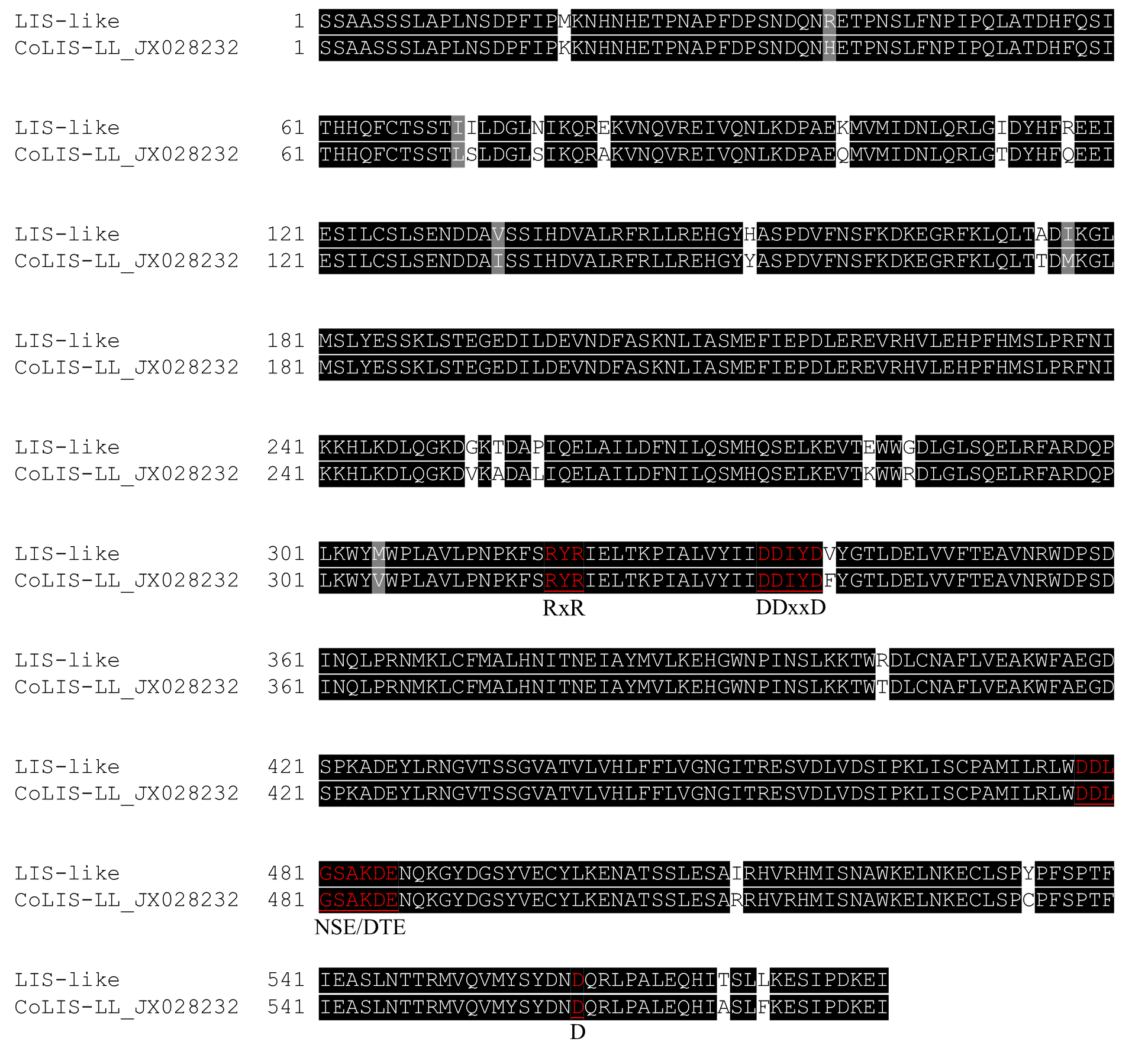


**Figure S4.** Amino acid sequence alignment of identified LIS-like in *C. camphora* with linalool synthase CoLIS-LL in *C. osmophloeum* (accession number: JX028232). Fully conserved amino acids are shaded in black and enzymatic motifs are highlighted in red.
